# Sex and gender differences and pharmacovigilance: a knot still to be untied

**DOI:** 10.3389/fphar.2024.1397291

**Published:** 2024-04-17

**Authors:** Liberata Sportiello, Annalisa Capuano

**Affiliations:** ^1^ Campania Regional Centre for Pharmacovigilance and Pharmacoepidemiology, University of Campania “Luigi Vanvitelli”, Naples, Italy; ^2^ Department of Experimental Medicine, University of Campania “Luigi Vanvitelli”, Naples, Italy

**Keywords:** adverse drug reaction, sex, gender differences, pharmacology, pharmacovigilance

## 1 Introduction

To date, the confusion between terms “sex” and “gender” seems to be less frequent in the scientific community. However, in medicine research it is still difficult to establish whether differences between women and men depend on the influence of sex, gender or both (Lapeyre-Mestre, 2019). Moreover, sex and gender interact with each other. The World Health Organization (WHO) defines gender medicine as the study of the influence of biological (defined by sex) and socio-economic, environmental and cultural (defined by gender) differences on the people’s health or disease status (World Health Organization, 2024). Over the last decades, the research in the gender medicine has significantly increased. Specifically, the attention to the gender in the field of pharmacological research can allow the development of personalized therapies, which aim at the centrality of the patient. This approach represents one of the innovative horizons on which biomedical research is focusing today. From this perspective, the gender pharmacology studies the sex and gender differences in pharmacological treatments in terms of effectiveness and safety. It carefully considers all physiological and non-physiological variables that could influence the drug response, in order to promote the equity and the appropriateness of treatments.

Women and men can differ in drug response due to sex-related key variables, including body weight, height, body surface area, fat mass, and plasma volume, which in turn depend on other relevant factors such as genes, hormones and age (Mauvais-Jarvis et al., 2021). These parameters influence pharmacokinetic and pharmacodynamic processes (Spoletini et al., 2012). The analysis of pharmacodynamic parameters is more complex than pharmacokinetic ones because it should be based on the demonstration that a drug produces different pharmacological effects in the two sexes. While the sex is static factor, the gender is a dynamic and modifiable process in permanent interaction with other characteristics. Therefore, the study of gender differences is more difficult (Lapeyre-Mestre, 2019). Thus, seeing the woman as a “variant” of the man is a cultural problem with distant roots and only in more recent years a global awareness has arisen. Despite this, men and women continue to be considered as unique in terms of healthcare management and therapeutic treatments (with rare exceptions).

In light of these brief considerations, we need a new strategy outline to better study the efficacy and safety profile of medicines in both sexes. Therefore, the purpose of this article is to discuss the critical points of clinical research should be improved in order to bring out sex and gender differences.

## 2 Differences between women and men in the enrollment in clinical trials

Women have been historically under-represented in drug and vaccine clinical trials. To date, more males than females still continue to be enrolled in clinical trials. However, it is difficult to establish the rate of women included into clinical studies. Mayor et al. conducted an analysis of US trials in common vascular diseases by using data from ClinicalTrials.gov from 2008 to 2020 (Mayor et al., 2022). They found the participation of women in these trials was low and has not enhanced over the considered time. The authors questioned whether the generalizability of scientific findings to women from recent trial results was efficient. In their opinion, a better understanding of the underlying causes for poor female trial participation is needed. Furthermore, even when women are included in clinical trials, sex (and not gender) is mostly considered in the description of participants’ sociodemographic characteristics, while the results are almost never analyzed by subgroups or reported separately for men and women (Mayor et al., 2022). Welch et al. conducted a study to evaluate whether sex and gender analysis was carried out in a sample of published Canadian randomized controlled trials (RCTs). On a total of 100 RCTs, the 98% of the studies included sex in the description of the socio-demographic characteristics of the enrolled patients, while only 6% conducted a subgroup analysis based on sex and only 4% reported sex-disaggregated data (Welch et al., 2017).

## 3 Differences between women and men in the use of medicines

Several studies investigated on differences in drug utilization between men and women. Loikas et al. performed a cross-sectional analysis of dispensed drugs in Sweden showing that women were dispensed more drugs than men and the widest sex difference in prevalence was found for antibiotics, thyroid therapy and antidepressants (Loikas et al., 2013). Manteuffel et al. conducted an analysis of pharmacy and medical claims in the United States, in order to evaluate the sex differences in medication use and adherence, and prescribing alignment with clinical guidelines. They found that women were significantly more likely than men to use one or more drugs. At the same time, women were less likely than men to be adherent to chronic treatments and to receive the pharmacological therapies and monitoring recommended by clinical guidelines (Manteuffel et al., 2014). Other evidence confirmed that also older women tend to utilize more medicines than men. In addition, some studies suggested that prescription of potentially inappropriate medications vary by gender (Alwhaibi and Balkhi, 2023).

If the use of medicines in women is higher than in men, it is important to understand the underlying reasons. Firstly, women seem to have more painful symptoms (e.g., migraines, musculoskeletal pains). Secondly, the physiological events in a woman’s life (e.g., menstruation, pregnancy, menopause) have been and are extremely medicalized. Thirdly, women pay more attention to her health conditions, especially in the case of chronic diseases (Ek, 2015). Moreover, despite their longer life expectancy (defined as “woman paradox”), women more get sicker than men. Several hypotheses have been proposed for sex differences in longevity (Van Oyen et al., 2013; Stephan et al., 2021; Phillips et al., 2023). However, it still needs further detailing the causes of these differences.

## 4 Differences between women and men in pharmacogenetics and pharmacogenomics

The pharmacogenetics, which is the study of inter-individual differences in drug response partially determined by genetic factors, has greatly increased until the sequencing of the human genome in the early 2000 s which lead to the metamorphosis of pharmacogenetics into pharmacogenomics (Auwerx et al., 2022). The latter aims at discovering genetic variants affecting drug response by probing the entire genome, as opposed to a few candidate loci. Its application has the power to help improve drug efficacy and avoid suspected adverse drug reactions (ADRs). It has been shown that many genetic polymorphisms present sex–gender specificity (Franconi and Campesi, 2014). If on one side evidence on sex disparity have been gained in terms of incidence, prognosis and treatment of several diseases (cardiovascular, cancer, neurodegenerative, bone homeostasis, infectious and painful), on the other side less is known about the interconnection between sex, genetic factors and drug safety. Some studies have found different drug efficacy and safety profiles between males and females, from cholesterol-lowering proprotein convertase subtilisin kexin type 9 (PCSK9) inhibitors to therapies for mental health and chronic pain management. An example where sex seems to modify the interplay between genetic factors and drug efficacy is the PCSK9-R46L variant. Based on this, the pharmacological inhibition of PCSK9 is resulted stronger in men compared to women, thus less women achieve target reductions in LDL-C levels compared to men (Corpas et al., 2024). A recent retrospective review of patients previously enrolled in the RIGHT10K (Right Drug, Right Dose, Right Time: Using Genomic Data to Individualize Treatment) database with major depressive disorder investigated on the risk of antidepressant interruption between males and females accounting for cytochrome P450 phenotype (fast, middle and slow categories). In this case, the results did not show a significant difference (Kosaski et al., 2023). On contrary, a prospective observational study was conducted on chronic non-cancer pain (CNCP) ambulatory patients and opioid use disorder (OUD), in which they underwent a opioid dose reduction and discontinuation. By analyzing sex differences and CYP2D6 phenotypes (poor, extensive and ultrarapid metabolizers), a tendency to a lower analgesic tolerability in females and lower quality of life in men was observed (Muriel et al., 2023). Therefore, sex may play a relevant role in the tolerability when opioid deprescribing. In line with the development and the use of genetic tools in routine medical practice with a great impact in healthcare (Gemmati et al., 2019), gender medicine with the prioritizing the role of sex/gender in physiological and pathological processes needs to follow the same process and become an established medical approach in all its fields.

## 5 Differences between women and men in the adverse drug reactions

To date it is known that the frequency of ADR is greater in women than in men, since female sex seems to be a risk factor for the development of ADRs (with a 1.5 to 1.7 times higher than in the male sex) (Rademaker, 2001; Franconi and Campesi, 2014). Women are not only at higher risk of hospitalization due to ADRs, but they are also more likely to discontinue treatment and, therefore, losing its potential benefit. Furthermore, the reporting of one or more ADRs includes the choice to connect the onset of signs or symptoms to a drug, and this evaluation is felt differently by women and men. Compared with men, women showed a greater interest in health issues. Many studies highlighted that the number of safety reports is more in women than in men. However, although women report more ADRs, men report more serious and fatal ADRs (Holm et al., 2017). These findings were found by Watson et al. by analyzing data collected within VigiBase, the WHO global database of individual case safety reports, between 1967 and 2018 (Watson et al., 2019). Women reported more ADRs (60.1%) than men (39.9%). The largest difference was observed in the age group of 18–44 years and could not be explained by hormonal contraceptive use. The proportion of serious and fatal reports was higher for male reports. Also Montastruc et al. analyzed the same database (VigiBase^®^) in a 10 year-period (2010–2019), focusing on fatal ADRs. They found that the risk of reporting fatal events was higher in males than in women and the most frequent drug classes involved were antineoplastic/immunomodulating drugs (Montastruc et al., 2021). Wabont et al. performed a disproportionality analysis of VigiBase^®^ on sex differences in serious ADRs in patients receiving immunotherapy, targeted therapy, or chemotherapy, between 1967 and 2022. Although they observed a higher report of ADRs for women (59.0%), less serious symptomatic ADRs are reported for women compared with men (Gemmati et al., 2019).

## 6 Discussion

Over the time, the research in medicine has showed significant differences between men and women in regard to the incidence, prevalence, severity and prognosis of several diseases (Gemmati et al., 2019), but probably less in terms of response to pharmacological treatments. This gap characterizes both efficacy and safety profile of medicines. Despite women are less enrolled in clinical trials, they seem to be more exposed to drugs throughout their lives and consequently they have more possibility of seeing ADRs.

The identification, reporting and evaluation of ADRs are important pharmacovigilance activities conducted to understand and prevent their occurrence (Sessa et al., 2015; Sportiello et al., 2016a; Rafaniello et al., 2016; Scisciola et al., 2022) However, the spontaneous reporting systems on ADRs are not internationally standardized (Bailey et al., 2016; Brabete et al., 2022). Safety reports rarely include gender data. Moreover, sex and gender are often used interchangeably. Taking into account that the quality of reporting is extremely relevant, the lack of data on sex, gender or other variables, such as age, also influences the interpretation of the results. However, the recent efforts by European Medicines Agency (EMA) are giving a positive contribute in order to standardize the procedures at European level (Santoro et al., 2017; European Medicines Agency, 2022). We are waiting for a global standardization!

Despite the limitations associated with the spontaneous reporting system, it can represent an immediate methodological approach for the evaluation of potential sex and gender differences in terms of drug safety (Ruggiero et al., 2020; Ruggiero et al., 2022; Ruggiero et al., 2023). However, the identification and evaluation of differences in ADRs between men and women are still poorly known and sometimes controversial. In fact, despite the frequency of ADR onset appears to be higher in women than in men, emerging data related to specific diseases or class of drugs sometimes highlighted different trend (Sportiello et al., 2016b; Capuano et al., 2020). Therefore, these findings need to be investigated in more detail.

Considering that many gaps are still in gender pharmacology, we are only at the beginning of this new era. Several researchers defined this era as gender and sex-Omics era. The omics-approach is a powerful tool to the discovery and identification of sex/gender-specific disease markers, novel drug-targets/therapeutic protocols, personalized laboratory tests and diagnostic procedures.

First of all, we should think to renew the entire process of pharmacological research in order to carry out optimal quality analysis on drug efficacy and safety and to highlight substantial sex and gender differences. Research in the gender pharmacology has important methodological gaps both in pre-marketing and post-marketing phases. In fact, guidelines are needed for analyzing data by sex and gender (by subgroups of patients or by disaggregating data) and for presenting the emerging results in more suitable manner. Therefore, the study protocols in clinical trials should be modified. First of all, a balanced inclusion of women and men as well as guideline-recommended sex-stratified analyses of all experienced adverse events in all phases of clinical trials is warranted. Revolutionary changes can also be made upstream. In fact, the use of gender-specific pre-clinical models can encourage the application of gender-oriented therapeutic protocols. This can accelerate the development of gender-specific drugs and the setting of gender-oriented and evidence-based guidelines (Gemmati et al., 2019).

Moreover, available safety data prove that sex and gender differences matter and should be examined not only in the clinical trials but also in the real world setting to improve the characterization of ADRs. A call to action is needed to incorporate sex-specific reporting into clinical practice. But there is more: sex and gender are different factors. Most of the evidence consider sex and not gender. To date, reporting systems of ADRs do not allow analysis by gender, but only by sex. Such systems should also be implemented. Therefore, evaluating sex- and gender-specific adverse events should be a priority in pharmacovigilance and pharmacoepidemiology studies. Lapeyre-Mestre et al. highlighted that women may experience different ADRs than men when treated with the same drugs, and that differences in ADR reporting are likely emphasized because prescribing and management practices are often related to the patient’s gender (Lapeyre-Mestre, 2019). Therefore, it is not enough to adjust data by sex in order to investigate potential differences between women and men in studies on the safety profile of drugs. However, in most cases, even only sex-specific data remains poor. Consequently, a wind of change in pharmacovigilance and pharmacoepidemiology studies can play a key role in increasing knowledge on these differences. Above all, it is crucial to consider this aspect from the beginning, namely, during the concept and the design of new studies. This could help to better understand sex-related (and even gender-related) ADRs and could promote the identification of sex-specific ADRs in many fields. We think that the analysis of sex and gender differences in the real world can contribute to the general understanding of the effects of these effect modifiers rather than confounders on safety of drugs. However, its feasibility is complex because often other factors and variables which in turn influence the final outcome have been ignored, sometimes interplaying with the sex and the multifactorial gender. Therefore, only accurate pre- and clinical investigations can direct pharmacovigilance and pharmacoepidemiology studies towards sex and gender.

## 7 Final considerations

Given that the research on sex and gender differences continues to gather increasing interest in the field of pharmacology and in pharmacovigilance, we hope that the emerging evidence on this topic encourage further research to highlight sex and gender differences. Even only this increasing attention can represent the first puzzle piece to start bridging the gap. Such improved knowledge could provide useful suggestions to governance for a better healthcare management of female and male patients and also to guide future research in this field.

In conclusion, the goal is not to obtain equal outcomes in women and men, but to achieve gender equity in the field of outcomes, offering all genders the best possible outcome (Franconi et al., 2007). In fact, considering sex and gender in pre-clinical, clinical and post-clinical studies in order to understand any unrevealed male and female difference can give the possibility to achieve a fully inclusive personalized medicine. Therefore, gender medicine represents the crucial assumption for achieving the personalized healthcare required in the third millennium (Gemmati et al., 2019).
